# Numerical Study of Entropy Generation in Fully Developed Turbulent Circular Tube Flow Using an Elliptic Blending Turbulence Model

**DOI:** 10.3390/e24020295

**Published:** 2022-02-19

**Authors:** Xianglong Yang, Lei Yang

**Affiliations:** 1 College of Civil and Transportation Engineering, Shenzhen University, Shenzhen 518060, China; xlyang@szu.edu.cn; 2 Key Laboratory for Resilient Infrastructures of Coastal Cities (MOE), Shenzhen University, Shenzhen 518060, China

**Keywords:** entropy generation, numerical study, circular tube, elliptic blending, turbulence model

## Abstract

As computational fluid dynamics (CFD) advances, entropy generation minimization based on CFD becomes attractive for optimizing complex heat-transfer systems. This optimization depends on the accuracy of CFD results, such that accurate turbulence models, such as elliptic relaxation or elliptic blending turbulence models, become important. The performance of a previously developed elliptic blending turbulence model (the SST k–ω–φ–α model) to predict the rate of entropy generation in the fully developed turbulent circular tube flow with constant heat flux was studied to provide some guidelines for using this class of turbulence model to calculate entropy generation in complex systems. The flow and temperature fields were simulated by using a CFD package, and then the rate of entropy generation was calculated in post-processing. The analytical correlations and results of two popular turbulence models (the realizable *k*–*ε* and the shear stress transport (SST) *k*–*ω* models) were used as references to demonstrate the accuracy of the SST k–ω–φ–α model. The findings indicate that the turbulent Prandtl number (Pr_t_) influences the entropy generation rate due to heat-transfer irreversibility. Pr_t_ = 0.85 produces the best results for the SST k–ω–φ–α model. For the realizable *k*–*ε* and SST *k*–*ω* models, Pr_t_ = 0.85 and Pr_t_ = 0.92 produce the best results, respectively. For the realizable *k*–*ε* and the SST *k*–*ω* models, the two methods used to predict the rate of entropy generation due to friction irreversibility produce the same results. However, for the SST k–ω–φ–α model, the rates of entropy generation due to friction irreversibility predicted by the two methods are different. The difference at a Reynolds number of 100,000 is about 14%. The method that incorporates the effective turbulent viscosity should be used to predict the rate of entropy generation due to friction irreversibility for the SST k–ω–φ–α model. Furthermore, when the temperature in the flow field changes dramatically, the temperature-dependent fluid properties must be considered.

## 1. Introduction

Due to the shortage of fossil energy and a desire for sustainable development, the demand for high-efficiency heat-transfer systems is increasing. Hence, much effort has been made to improve the efficiency of heat-transfer systems. However, much of this work is based on the first law of thermodynamics, which can provide a lot of useful information but does not provide optimal conditions. The entropy generation analysis (EGA), which is based on the second law of thermodynamics, is a powerful tool for heat-transfer-process optimization.

The EGA has been used in designing and optimizing thermal systems since its introduction by Bejan [1,2]. However, because earlier attempts were primarily theoretical, they could only deal with simple geometry configurations and flow conditions. Furthermore, some empirical correlations had to be introduced, because obtaining local information is difficult [3,4,5]. Flow and temperature fields can be obtained with the advancement of computational fluid dynamics (CFD), allowing the local entropy production to be computed. It is important for optimizing complex systems, because precise policy implementation is possible using local information. CFD has been used as a tool to evaluate the entropy generation and further optimize heat-transfer systems by a few researchers. Because the flow in many heat-transfer systems is turbulent, the turbulence characteristics must be considered. The Reynolds averaged Navier–Stokes (RANS)-based *k*–*ε* and *k*–*ω* models are the most commonly used turbulence models for calculating entropy generation. To avoid verbosity, only a few of them are discussed here. Shuja et al. [6] used four turbulence models to calculate the local entropy generation in a fluid jet impinging on a heated wall: the standard *k*–*ε* model, the low Reynolds number (Re) *k*–*ε* model, and two Reynolds stress models. Mwesigye et al. [7] used the realizable *k*–*ε* model to perform a numerical analysis of entropy generation in a parabolic trough receiver. Saqr et al. [8] investigated the entropy generation in a swirl pipe flow, using the realizable *k*–*ε* model. Wang et al. [9] modeled the flow around the NACA0012 airfoil and discussed the effect of turbulence models on entropy generation, using five turbulence models, namely the standard *k*–*ε* model, renormalization group (RNG) *k*–*ε* model, standard *k*–*ω* model, shear stress transport (SST) *k*–*ω* model, and Spalart–Allmaras (S–A) model. Ghorani et al. [10] used the entropy-generation-rate method with the SST *k*–*ω* model to investigate the flow within a centrifugal pump in a reverse mode. Yu et al. [11] investigated the detailed characteristics of the entropy production rate by simulating the flow in the Francis turbine, using the SST *k*–*ω* model. They also studied the effect of the mass flow rate on the entropy generation in the Francis turbine [12]. Pidaparthi et al. [13] analyzed and optimized the heat-transfer performance in a tube with internal helical fins by using EAG with the realizable *k*–*ε* model. Heat-transfer optimization of nanofluids by using EGA has also received attention. For example, Bianco et al. used the standard *k*–*ε* model to investigate the turbulent convection of Al_2_O_3_–water nanofluid inside a circular section tube subjected to constant heat flux [14] and constant wall temperature [15]. Mwesigye and Huan [16] investigated the turbulent nanofluid flow in a circular tube, using the entropy-generation-minimization method, with the help of a realizable *k*–*ε* model. Rashidi et al. [17] compared the single- and two-phase modeling approaches for heat-transfer performance in forced convection flow of TiO_2_-water nanofluid through EGA with the standard *k*–*ε* model. Bazdidi-Tehrani et al. [18] investigated the entropy generation in a CuO-water nanofluid flowing in a vertical duct, using the SST *k*–*ω* model. The SST *k*–*ω* model was also used by Fadodun et al. [19] to investigate the entropy production rate in a nanofluid flowing through an inwardly corrugated pipe in a turbulent flow regime. Bahiraei et al. [20] investigated the entropy generation in a nanofluid flow in a shell-and-tube heat exchanger with the RNG *k*–*ε* model.

Obviously, the accuracy of the flow and temperature fields predicted in CFD simulations affect the computing accuracy of local entropy generation. It is well-known that the *k*–*ε* and *k*–*ω* models have limitations when used in complex systems. Another type of RANS-based turbulence model, the elliptic relaxation model, or the elliptic blending model, which has been shown to outperform the *k*–*ε* and *k*–*ω* models in some complex systems, such as the 2D asymmetric diffuser flow, the sudden expansion pipe flow and the impinging jet flow [21,22], is expectedly more attractive for calculating local entropy generation. However, the application of such turbulence models in research on entropy generation has not been found in the literature, and this aspect has yet to be attempted.

This study investigated the performance of an elliptic-blending turbulence model developed in our previous work [22] to predict the local entropy generation rate in fully developed turbulent flows in a circular tube with constant heat flux. The purpose is to provide some guidelines for using this class of turbulence model to calculate entropy generation in complex systems. The accuracy of the elliptic-blending turbulence model was demonstrated by comparing it to the analytical correlations and the results of two other popular turbulence models (the realizable *k*–*ε* and the SST *k*–*ω* models). The computational method for entropy generation rate, the effect of the turbulent Prandtl number (Pr_t_), the effect of large wall-fluid temperature difference, and the effect of the temperature-dependent fluid properties were investigated. The findings—for example, the Pr_t_ should be chosen as 0.85, the method that incorporates the effective turbulent viscosity should be used to predict the rate of entropy generation due to friction irreversibility, and the temperature-dependent fluid properties should be considered when the temperature difference is large—can guide the application of the elliptic blending turbulence model to complex problems.

The numerical modeling is briefly described in the next section. Then, in the third section, the results are presented and discussed in detail. Finally, the conclusions are presented.

## 2. Numerical Modeling

**Figure 1:**
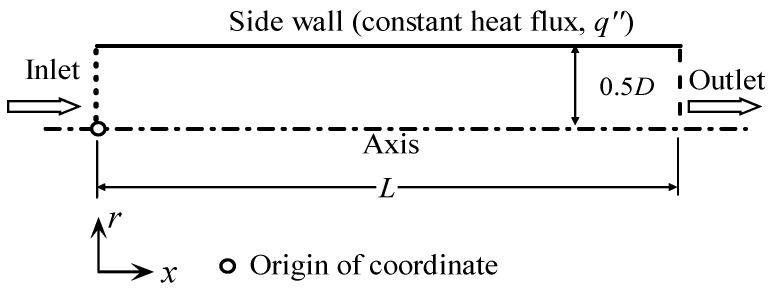
Schematic of the geometry and boundary conditions of the circular tube flow.

The problem under consideration is fully developed turbulent flow in a straight circular tube (diameter, *D*, and length, *L*) with a uniform constant heat flux ($q^{\prime \prime }$) on the sidewall. Because of the axial symmetry of the problem under consideration, an axisymmetric computational model was constructed (Figure 1).

### 2.1. Governing Equations

The flow under consideration in this study is steady, incompressible, and turbulent. Turbulence was handled by using the Reynolds averaging method, yielding the steady RANS equations given as follows:

(1) $$\frac{\partial }{\partial x_{i}}(\rho u_{i})=0,$$

(2) $$\frac{\partial }{\partial x_{j}}\left(\rho u_{i}u_{j}\right)=-\frac{\partial p}{\partial x_{i}}+\frac{\partial }{\partial x_{j}}\left[\mu \left(\frac{\partial u_{i}}{\partial x_{j}}+\frac{\partial u_{j}}{\partial x_{i}}\right)-\rho \bar{u_{i}^{\prime }u_{j}^{\prime }}\right]$$

For linear eddy viscosity turbulence models, the turbulent stresses are linked to the strain rate by using the following:

(3) $$-\rho \bar{u_{i}^{\prime }u_{j}^{\prime }}=\mu_{t}\left(\frac{\partial u_{i}}{\partial x_{j}}+\frac{\partial u_{j}}{\partial x_{i}}\right)-\frac{2}{3}\rho k\delta_{ij}$$

For incompressible flow, after using the Boussinesq approximation, the governing equation of energy can be simplified to the mean temperature (*T*) equation, as given by the following:

(4) $$\frac{\partial \rho T}{\partial t}+\frac{\partial }{\partial x_{i}}\left({\rho u}_{i}T\right)=\frac{\partial }{\partial x_{j}}\left[\left(\frac{\mu }{\mathrm{Pr}}+\frac{\mu_{t}}{{\mathrm{Pr}}_{t}}\right)\frac{\partial T}{\partial x_{j}}\right].$$

The turbulence model yields the eddy viscosity, *μ_t_*. The authors’ previously developed SST-incorporated elliptic-blending turbulence model (denoted as SST k–ω–φ–α model) was used. The model comprises four equations for the turbulent kinetic energy, *k*; specific dissipation rate, *ω*; wall-normal turbulent anisotropy, φ; and elliptic variable, *α*, respectively. The following are the model equations.

(5) $$\frac{\partial \rho k}{\partial t}+\frac{\partial }{\partial x_{i}}\left(\rho u_{i}k\right)=G_{k}-\rho f_{k}\beta^{*}k\omega +\frac{\partial }{\partial x_{j}}\left[\left(\frac{\mu }{2}+\sigma_{k}\mu_{t}\right)\frac{\partial k}{\partial x_{j}}\right],$$

(6) $$\frac{\partial \rho \omega }{\partial t}+\frac{\partial }{\partial x_{i}}\left(\rho u_{i}\omega \right)=f_{\omega }\gamma \frac{\omega }{k}G_{k}-\rho \beta \omega^{2}+C_{D}+\frac{\partial }{\partial x_{j}}\left[\left(\frac{\mu }{2}+\sigma_{\omega }\mu_{t}\right)\frac{\partial \omega }{\partial x_{j}}\right],$$

(7) $$\frac{\partial \rho \varphi }{\partial t}+\frac{\partial }{\partial x_{i}}\left(\rho u_{i}\varphi \right)=\left(1-\alpha^{p}\right)\rho f_{wall}+\alpha^{p}\rho f_{\mathrm{hom}}-\frac{\varphi }{k}G_{k}+\frac{2}{k}\mu_{t}\sigma_{k}\frac{\partial \varphi }{\partial x_{j}}\frac{\partial k}{\partial x_{j}}+\frac{\partial }{\partial x_{j}}\left[\left(\frac{\mu }{2}+\sigma_{\varphi }\mu_{t}\right)\frac{\partial \varphi }{\partial x_{j}}\right],$$

(8) $$0=\frac{1-\alpha }{L^{2}}+\frac{\partial }{\partial x_{j}}\left(\frac{\partial \alpha }{\partial x_{j}}\right).$$

The eddy viscosity is defined by a blending formulation:

(9) $$\mu_{t}=\left(1-\alpha^{p}\right)C_{\mu }\rho \varphi k\min(T,T_{\lim})+\alpha^{p}\frac{a_{1}\rho k}{\max\left(a_{1}\omega ,F_{2}S\right)}$$

The model’s details are not provided here for the sake of brevity, and the interested reader is directed to Yang et al. [22]. Another two popular turbulence models, the realizable *k*–*ε* model [23] and the SST *k*–*ω* model [24], were also used for comparison.

### 2.2. Entropy Generations

In order to facilitate understanding, it is necessary to first declare the meaning of the superscripts on the rate of entropy generation, *S*, used in the following: ${\left(\cdot \right)}^{\prime }$ means the quantity per unit length, ${\left(\cdot \right)}^{\prime \prime }$ means the quantity per unit area, and ${\left(\cdot \right)}^{\prime \prime \prime }$ means the quantity per unit volume.

The volumetric rate of entropy generation in viscous flow with convective heat transfer can be written as follows [3]:

(10) $$S_{gen}^{\prime \prime \prime }=S_{gen,f}^{\prime \prime \prime }+S_{gen,h}^{\prime \prime \prime },$$

where the first and second terms on the right-hand side are the local volumetric rate of entropy generation due to friction irreversibility and heat transfer irreversibility, respectively. Both terms can be expressed in cylindrical coordinates for circular tube flow [3]:

(11) $$S_{gen,f}^{\prime \prime \prime }=\frac{\mu }{T}\left\{2\left[{\left(\frac{\partial u_{r}}{\partial r}\right)}^{2}+{\left(\frac{u_{r}}{r}\right)}^{2}+{\left(\frac{\partial u_{x}}{\partial x}\right)}^{2}\right]+{\left(\frac{\partial u_{x}}{\partial r}+\frac{\partial u_{r}}{\partial x}\right)}^{2}\right\}$$

and

(12) $$S_{gen,h}^{\prime \prime \prime }=\frac{\lambda }{T^{2}}\left[{\left(\frac{\partial T}{\partial r}\right)}^{2}+{\left(\frac{\partial T}{\partial x}\right)}^{2}\right],$$

where *T* is the local mean temperature of flow, and *µ* and *λ* are the molecular dynamic viscosity and the molecular thermal conductivity of the fluid, respectively. The volumetric rate of entropy generation is affected by absolute temperature, viscosity, thermal conductivity, and local spatial gradients of velocity and temperature. These quantities can be easily obtained by using CFD.

The two equations above are valid for laminar flow. For turbulent flow, there are two different processing methods when the mean values of the quantities are used to calculate the rate of entropy generation. One can still use Equations (11) and (12), but *µ* and *λ* must be replaced with effective viscosity (*µ* + *µ_t_*) and effective thermal conductivity (*λ* + *λ_t_*), respectively, where *µ_t_* is the eddy viscosity and *λ_t_* is the turbulent thermal conductivity [25,26]. Moreover, Equations (11) and (12) become the following:

(13) $$S_{gen,f}^{\prime \prime \prime }=\frac{\mu +\mu_{t}}{T}\left\{2\left[{\left(\frac{\partial u_{r}}{\partial r}\right)}^{2}+{\left(\frac{u_{r}}{r}\right)}^{2}+{\left(\frac{\partial u_{x}}{\partial x}\right)}^{2}\right]+{\left(\frac{\partial u_{x}}{\partial r}+\frac{\partial u_{r}}{\partial x}\right)}^{2}\right\},$$

(14) $$S_{gen,h}^{\prime \prime \prime }=\frac{\lambda +\lambda_{t}}{T^{2}}\left[{\left(\frac{\partial T}{\partial r}\right)}^{2}+{\left(\frac{\partial T}{\partial x}\right)}^{2}\right].$$

Another method was developed by Kock and Herwig [27]. Using the time-averaging process gives $S_{gen,f}^{\prime \prime \prime }$ as follows:

(15) $$S_{gen,f}^{\prime \prime \prime }=\frac{\mu }{T}\left\{2\left[{\left(\frac{\partial u_{r}}{\partial r}\right)}^{2}+{\left(\frac{u_{r}}{r}\right)}^{2}+{\left(\frac{\partial u_{x}}{\partial x}\right)}^{2}\right]+{\left(\frac{\partial u_{x}}{\partial r}+\frac{\partial u_{r}}{\partial x}\right)}^{2}\right\}+\frac{\rho \varepsilon }{T}.$$

For $S_{gen,h}^{\prime \prime \prime }$, Equation (14) still holds. Equation (15) appears to have a natural advantage for *k*–*ε* models because the turbulence dissipation rate *ε* is obtained directly [28]. However, it could pose challenges to turbulence models where the accurate *ε* is difficult to obtain.

The following integral can be used to calculate the total rate of entropy generation in a fluid with volume, *V*:

(16) $$S_{gen}=\int_{V}S_{gen}^{\prime \prime \prime }dV=\int_{V}(S_{gen,f}^{\prime \prime \prime }+S_{gen,h}^{\prime \prime \prime })dV=S_{gen,f}+S_{gen,h}.$$

where

(17) $$S_{gen,f}=\int_{V}S_{gen,f}^{\prime \prime \prime }dV,$$

(18) $$S_{gen,h}=\int_{V}S_{gen,h}^{\prime \prime \prime }dV.$$

For fully developed turbulent circular tube flow, Bejan [3] gave the analytical expressions for the rate of entropy generation per unit length as follows:

(19) $$S_{gen,f}^{\prime }=\frac{8{\dot{m}}^{3}f}{\pi^{2}\rho^{2}T_{ave}^{2}D^{5}},$$

(20) $$S_{gen,h}^{\prime }=\frac{{q^{\prime }}^{2}}{\pi \lambda T_{ave}^{2}Nu}.$$

where *T_ave_* is the average temperature in the domain, *f* is the friction factor, $\dot{m}\;$is the mass flow rate, $q^{\prime }$ is the heat flux per unit length, and *Nu* is the Nusselt number.

For *Nu*, the widely used correlations are the Gnielinski’s correlation [29]:

(21) Nu=(f/8)(Re−1000)Pr1+12.7(f/8)0.5(Pr2/3−1),

and the Dittus–Boelter correlation [29]:

(22) Nu=0.023Re0.8Pr0.4.

where Pr is the molecular Prandtl number. In Gnielinski’s correlation (Equation (21)), the friction factor is given by the Petukhov’s correlation [29]:

(23) f=(0.79lnRe−1.64)−2.

This correlation can also be used in Equation (19) to calculate $S_{gen,f}^{\prime }$.

### 2.3. Numerical Procedures

The CFD package Ansys Fluent (17.0) was used for all computations. In addition, the User-Defined Function functionality was used to implement the SST k–ω–φ–α model. The four turbulence variables, *k*, ω, φ, and α, were solved as User-Defined Scalar (UDS) quantities. The production and dissipation terms in these equations were added as “sources” to the transport equations, and the diffusivities of these variables were assigned accordingly. The SST *k*–*ω* and realizable *k*–*ε* models are pre-coded turbulence models available in Ansys Fluent that can be used directly.

The governing equations were solved by using the pressure-based Coupled algorithm. The second-order upwind scheme was used to discretize the convection terms in the momentum, energy, and turbulence equations. The velocity–pressure coupling process was handled by the Coupled algorithm, which solves the momentum and continuity equations in a closely coupled manner and can improve the rate of solution convergence significantly when compared to the segregated algorithm (such as the SIMPLE algorithm). The gradients and derivatives were evaluated by using the least-squares cell-based method. Furthermore, it should be noted that, in the current study, a double-precision solver was required to obtain a stable and accurate solution.

**Figure 2:**
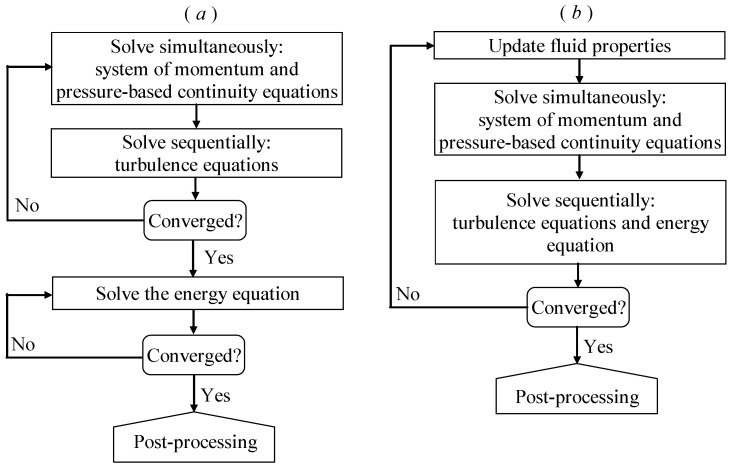
Overview of the solution procedures: (**a**) for constant fluid properties and (**b**) for temperature-dependent fluid properties.

Figure 2 shows the solution procedures used in the present paper. For the cases considered in Section 3.1, Section 3.2, Section 3.3 and Section 3.4 (using the procedure as Figure 2a), the thermal properties of the materials were assumed to be temperature independent, allowing the governing equations for fluid flow and the energy equation to be solved independently. The isothermal fluid flow was solved first, and then the energy equation was solved by using the converged solution of the fluid flow. For the cases considered in Section 3.5 (using the procedure as Figure 2b), the temperature-dependent thermal properties of the fluid were considered, so that the governing equations for fluid flow and the energy equation were solved continuously in each iteration. After obtaining convergent flow and temperature fields, post-processing was performed to obtain the desired quantities, such as the Nusselt number, friction factor, and rate of entropy generation.

### 2.4. Computational Grid and Boundary Conditions

The computational domain (as shown in Figure 1) was meshed by using a Cartesian grid. In the vertical direction of the wall (*r*-direction), to capture high resolutions of variables close to the wall, the successive ratio mesh was used. In the streamwise direction (*x*-direction), the cells were distributed uniformly. Several different grids were designed to examine the mesh convergence results, and then we decided upon the grid used in the present work. In the test cases, the successive ratio and the number of nodes in the *x*-direction (*N_x_*) kept being constants, as 1.05 and 100, respectively. The number of nodes in the *r*-direction (*N_r_*) was different. Table 1 shows the results computed by using the SST k–ω–φ–α model with temperature-independent fluid properties. It was clear that, when *N_r_* increases from 150 to 200, the change of the calculated parameters, *f*, *Nu*, $S_{gen,h}^{\prime }$,$\;\mathrm{and}\;S_{gen,f}^{\prime }$, whose reference values were evaluated by Equations (19)--(21), and (23), respectively, was small. Therefore, the mesh resolution of 200×100 was selected for the present work.

Figure 1 also depicts a schematic of the problem’s boundary conditions. Given that the turbulent flow in a circular tube is fully developed, it is appropriate to construct a periodic boundary condition for the inlet and outlet. The mass flow rate and temperature were specified at the inlet. To match the Re, the mass flow rate was adjusted. Throughout this study, the inlet temperature was 293.15 K. Constant heat flux was specified at the wall for the energy equation, and the no-slip condition was used for fluid flow. The wall treatments varied depending on the turbulence model. Specifically, for the SST k–ω–φ–α model, the conditions *u_i_* = 0, *k* = 0, φ = 0, *α* = 0, and $\omega =3\nu /\left(\beta_{1}y_{1}^{2}\right)$ were specified [22]. For the SST *k*–*ω* model, an automatic near-wall treatment method was used [30]. The enhanced wall-treatment method was used for the realizable *k*–*ε* model [31].

### 2.5. Data Reduction

The results were obtained through post–processing. The Nusselt number, friction factor, and entropy generation rate per unit length were the relevant parameters used in the analysis. They were computed as follows.

The Nusselt number is denoted by the following:

(24) $$Nu(x)=\frac{h(x)D}{\lambda }$$

where

(25) $$h(x)=\frac{q^{\prime \prime }}{T_{w}(x)-T_{m}(x)},$$

(26) $$T_{m}(x)=T_{in}+\frac{q^{\prime \prime }\pi Dx}{\dot{m}c_{p}}$$

where *h*(*x*) is the local convective heat-transfer coefficient; *q*” is the heat flux; *x* is the distance from inlet; *c_p_* is the specific heat of the fluid; and *T_in_*, *T_w_*, and *T_m_* are the inlet temperature, wall temperature, and bulk temperature, respectively. The average temperature *T_ave_* in Equations (19) and (20) is calculated as [14]

(27) $$T_{ave}=\frac{T_{in}-T_{m}(L)}{\ln(T_{in}/T_{m}(L))}.$$

The friction factor is represented by the following:

(28) $$f=\frac{8\tau_{w}}{\rho U_{b}^{2}}.$$

where $\tau_{w}$ is the wall shear stress, and *U_b_* is the bulk velocity. Given that the aspect ratio of the computational region (*L*/*D*) is small in this work, the rates of entropy generation per unit length were calculated by using Equations (17) and (18) as follows:

(29) $$S_{gen,f}^{\prime }=S_{gen,f}/L,$$

(30) $$S_{gen,h}^{\prime }=S_{gen,h}/L.$$

## 3. Results and Discussion

In present study, the working fluid is water, whose temperature-independent thermal properties at 293.15 K (used in Section 3.1, Section 3.2, Section 3.3 and Section 3.4) are listed in Table 2. For various purposes, three circular tubes with different cross-sectional areas (*A_c_*) of 0.000005 m^2^, 0.0005 m^2^, and 0.05 m^2^; five Reynolds numbers, 10,000, 30,000, 50,000, 70,000, and 100,000; and different heat fluxes, ranging from 500 to 30,000 W/m^2^, were used. The main concern in this paper is the rate of entropy generation per unit length, which was obtained by averaging along the flow direction (Equations (29) and (30)). To achieve accurate results, it requires that the physical quantity in the computational region cannot change too much along the flow direction. Consequently, a value of 0.5 for the aspect ratio (*L*/*D*) was selected for all tubes considered in this study. Table 3 lists other parameters used in the following sections. For Section 3.1, Section 3.2 and Section 3.3, the motivation for using different heat fluxes for different tubes was to ensure that the wall-bulk temperature difference (ΔT) in all tubes was small enough when compared to the bulk flow temperature (*T_m_*), and then the results could be compared with the analytical expressions of Bejan [3] (Equations (19) and (20)), in which the ratio of ΔT to *T_m_* was small and, thus, ignored. However, we discovered that, when a large heat flux (for example, 50,000 W/m^2^) is applied to a large tube (for example, *A_c_* = 0.05 m^2^), the computed rate of entropy generation deviates from the analytical expressions at a low Re. Mwesigye and Huan [16] discovered the same phenomenon. They reasoned that the large error was caused by the incorrect correlations used for *Nu* and *f*. However, we discovered in this study that ΔT is too large to be ignored in this case, and the condition that should be satisfied in Bejan’s analytical expression is violated.

The solution convergence history is influenced by the relaxation factors and the initial conditions. In present work, the relaxation factors for the four turbulent quantities of the SST k–ω–φ–α model were set to be 0.8, and the default values of the relaxation factors in the Ansys Fluent software were used for other quantities. The initial values (denoted by subscript 0) of the variables were set as follows: the turbulence intensity, $I_{0}=0.03$; the axial velocity, u0=μReρD; the radial velocity, $v_{0}=0,$ $k_{0}=1.5{\left(u_{0}I_{0}\right)}^{2}$, $\omega_{0}=\frac{k_{0}^{0.5}}{0.0383D}$, $\varepsilon_{0}=\frac{k_{0}^{1.5}}{0.426D}$, $\varphi_{0}=0.3,\;\mathrm{and}\;\alpha_{0}=1.0.$

Based on same computer hardware (24 CPU cores with 3.1 GHz frequency and 128 GB RAM), the computational efficiency was compared by using a case with constant fluid properties and *A_c_* = 0.05 m^2^, Re = 100,000, and *q*″ = 500 W/m^2^. The SST k–ω–φ–α, SST *k*–*ω*, and realizable *k*–*ε* models spent about 45, 32 and 28 min CPU time to obtain a convergent solution, respectively. The reason that the SST k–ω–φ–α model takes more computation time is that it has two more governing equations and more complex source terms.

### 3.1. Validation of the Friction Factor and Nusselt Number

**Figure 3:**
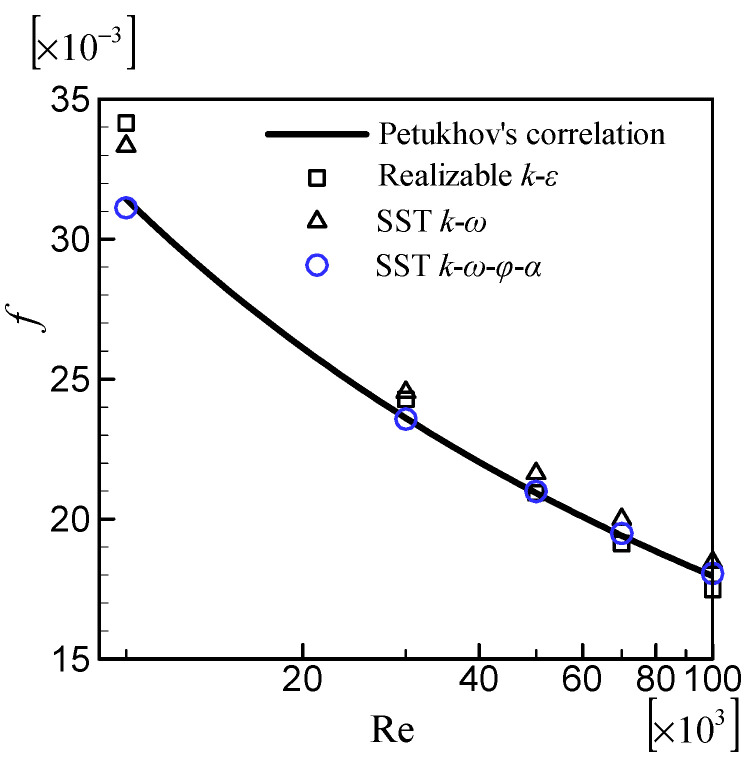
Comparison of the friction factors with Petukhov’s correlation.

The friction factor was used to validate the hydrodynamic performance of the turbulence model. Figure 3 compares friction factors calculated by various turbulence models with Petukhov’s correlation [29]. The SST k–ω–φ–α model achieves an excellent agreement for all Re. The SST *k*–*ω* model and the realizable *k*–*ε* model both predict good results at high Re but overestimate the friction factor at low Re. When the Re is lower, the deviation is greater. This is not surprising given that both the SST *k*–*ω* model and the realizable *k*–*ε* model we used in present work are developed for high Re flow and do not include any low Re corrections, whereas the SST k–ω–φ–α model is developed for low and high Re flow and includes some low Re corrections.

The Nusselt number was used to validate the heat-transfer performance. It should be noted that the Pr_t_ in Equation (4) affects the temperature solution, which, in turn, affects other quantities, such as the Nusselt number, effective thermal conductivity, and rate of entropy generation. Because the influence factors are so complex and not fully understood, Pr_t_ is difficult to derive from theory. In CFD applications, Pr_t_ is typically treated by using an empirical formula or as a constant determined by the slope of the temperature curve in the log region [32]. Even in simple wall shear flows, there is no universal constant value for Pr_t_. For example, the Pr_t_ ranges between 0.73 and 0.92 for airflow with Pr = 0.71 [32]. Furthermore, different turbulence models may necessitate different Pr_t_, due to different temperature profile predictions. Three constant values, 0.73, 0.85, and 0.92, were used to evaluate the effect of Pr_t_ on heat-transfer performance.

**Figure 4:**
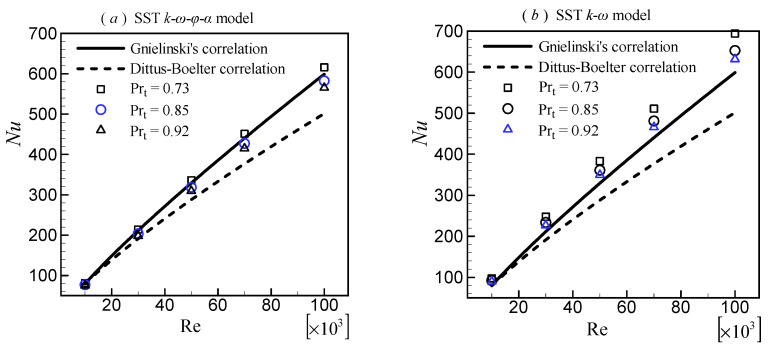

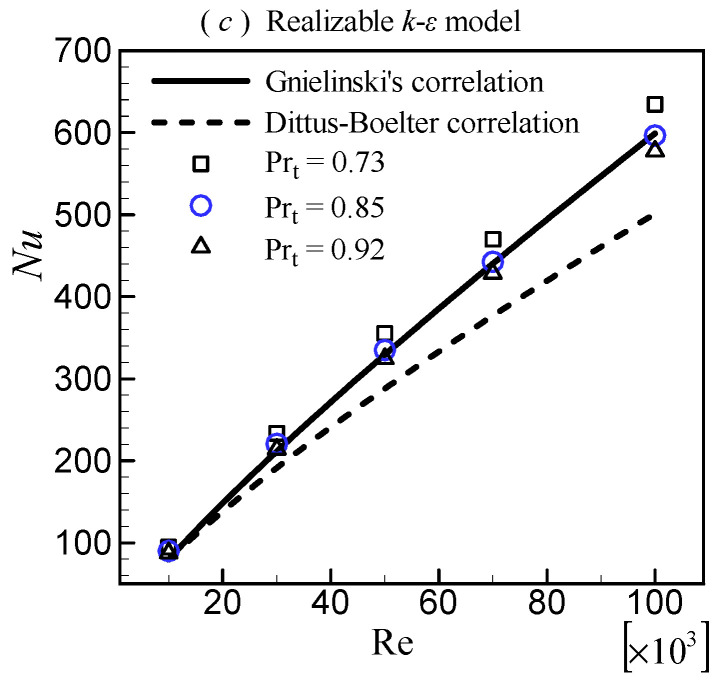
Effects of the turbulent Prandtl number on the Nusselt number.

The computed *Nu* by different Pr_t_ was compared with the Gnielinski’s correlation (Equation (21)) and the Dittus–Boelter correlation (Equation (22)). Figure 4 depicts the results. It is clear that a lower Pr_t_ leads to higher *Nu* for all turbulence models. All computed results deviate from the Dittus–Boelter correlation to a large extent. The results for the SST k–ω–φ–α model (Figure 4a) with Pr_t_ = 0.73 and 0.85 are in good agreement with Gnielinski’s correlation. The results with Pr_t_ = 0.92 deviate slightly. All results for the SST *k*–*ω* model (Figure 4b) deviate from Gnielinski’s correlation. The result with Pr_t_ = 0.92 is the best among them. The result with Pr_t_ = 0.85 for the realizable *k*–*ε* model (Figure 4c) is in good agreement with Gnielinski’s correlation. Pr_t_ = 0.73 overestimates the *Nu*. Pr_t_ = 0.92; however, underpredicts it.

Overall, the computed results for all three turbulence models agree better with the Gnielinski’s correlation than the Dittus–Boelter correlation, so the Gnielinski’s correlation was used where the correlation of *Nu* is required.

### 3.2. Rate of Entropy Generation Due to Heat Transfer Irreversibility

**Figure 5:**
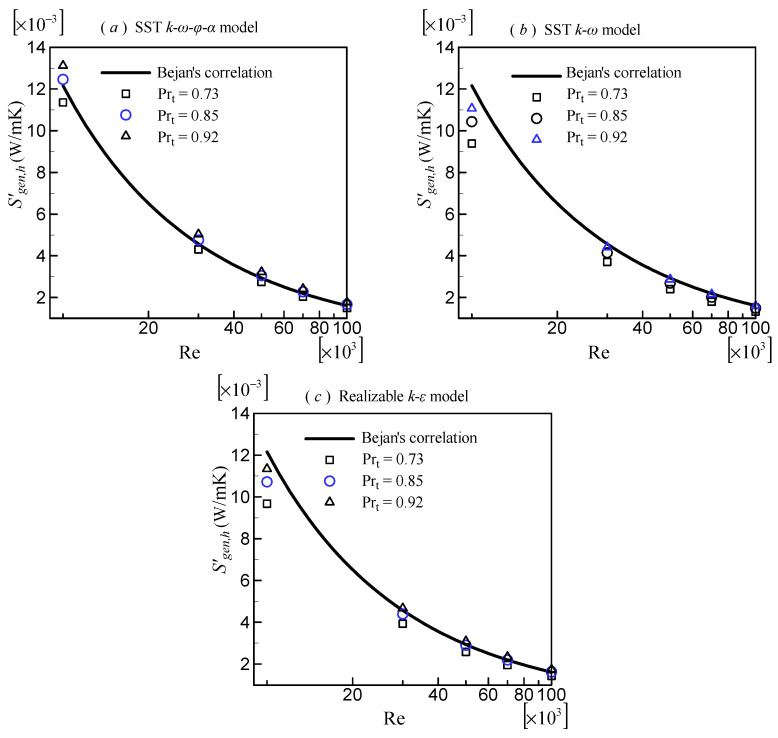
Effects of the turbulent Prandtl number on $S_{gen,h}^{\prime }$.

According to Equation (20), the rate of entropy generation per unit length due to heat transfer irreversibility, $S_{gen,h}^{\prime }$, is dependent on *T_ave_* and *Nu*, which is, in turn, dependent on Pr_t_. Consequently, $S_{gen,h}^{\prime }\;$will be affected by Pr_t_. Because the effect of Pr_t_ on $S_{gen,h}^{\prime }$ is similar for circular tubes of various sizes, only the results for *A_c_* = 0.000005 m^2^ were provided. Figure 5 depicts the variation of $S_{gen,h}^{\prime }$ with respect to Re computed by various turbulence models with varying Pr_t_. It is self-evident that a smaller Pr_t_ results in a smaller$\;S_{gen,h}^{\prime }$. This is the inverse of the effect of Pr_t_ on *Nu*. It is simple to understand because $S_{gen,h}^{\prime }$ is inversely proportional to *Nu*. When comparing the SST k–ω–φ–α model (Figure 5a) with the Bejan’s correlation (Equation (20)), we see that the result with Pr_t_ = 0.85 agrees very well. Pr_t_ = 0.73 overpredicts but Pr_t_ = 0.92 underpredicts $S_{gen,h}^{\prime }$. Comparing the SST *k*–*ω* model (Figure 5b) with the Bejan’s correlation, we see that all values of Pr_t_ underpredict$\;S_{gen,h}^{\prime }$, but the degree of deviation decreases as Re increases. The result with Pr_t_ = 0.92 is the best of the three Pr_t_. The results with Pr_t_ = 0.85 for the realizable *k*–*ε* model (Figure 5c) agree with the Bejan’s correlation for high Re. However, for low Re (Re = 10,000), the result with Pr_t_ = 0.92 is better. Based on this finding, considering together the effect of Pr_t_ on *Nu*, the Pr_t_ that yields good *Nu* and $S_{gen,h}^{\prime }$ was chosen in the subsequent analysis of each turbulence model, i.e., 0.85, 0.92, and 0.85 for the SST k–ω–φ–α model, SST *k*–*ω* model, and realizable *k*–*ε* model, respectively.

**Figure 6:**
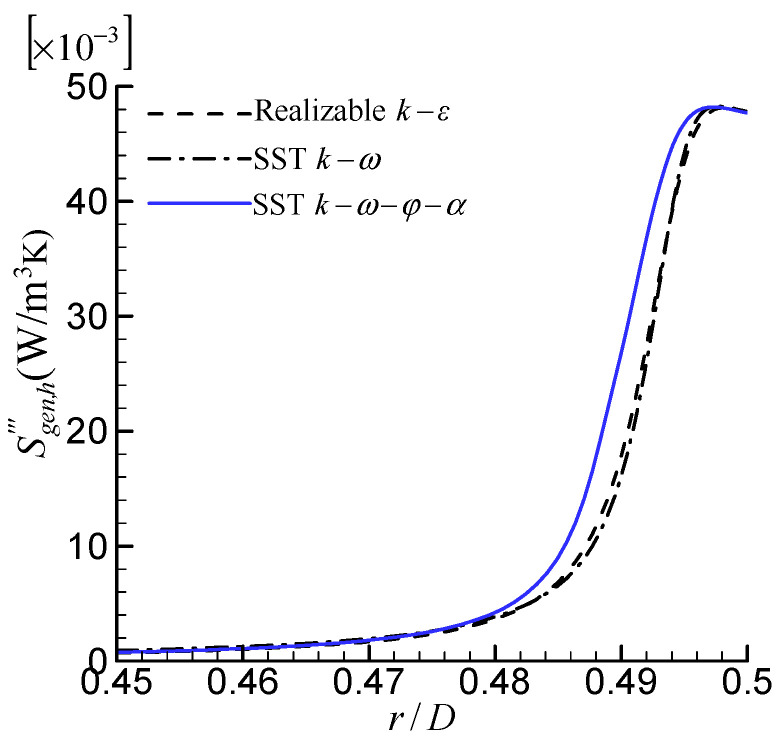
Comparison of local distribution of $\;S_{gen,h}^{\prime \prime \prime }$.

The local distribution of the rate of entropy generation per unit volume due to heat-transfer irreversibility, $S_{gen,h}^{\prime \prime \prime }$, along the radial direction at Re = 10,000 is shown in Figure 6. It can be seen that $S_{gen,h}^{\prime \prime \prime }$ mainly occurs in the near-wall region. It is not surprising because, in the near-wall region, the temperature gradient is large. The distributions of $S_{gen,h}^{\prime \prime \prime }$ predicted by the SST *k*–*ω* and realizable *k*–*ε* models are very close. The $S_{gen,h}^{\prime \prime \prime }$ predicted by the SST k–ω–φ–α model is larger than the other two.

### 3.3. Rate of Entropy Generation Due to Friction Irreversibility

As mentioned in Section 2.2, there are two methods in CFD applications for calculating the entropy generation due to friction irreversibility,$\;S_{gen,f}^{\prime }$. The first uses turbulent viscosity (Equation (13), denoted as Method 1), and the second uses the turbulent dissipation rate (Equation (15), denoted as Method 2). For the *k*–*ε* model series, Method 2 is natural. However, for other turbulence models in which *ε* is not easily determined, Method 2 may encounter difficulty. For example, in the SST k–ω–φ–α and SST *k*–*ω* models, although $\varepsilon =\beta^{*}k\omega$ was used when the models were developed, it does not hold everywhere due to some simplifications, modifications, and corrections.

**Figure 7:**
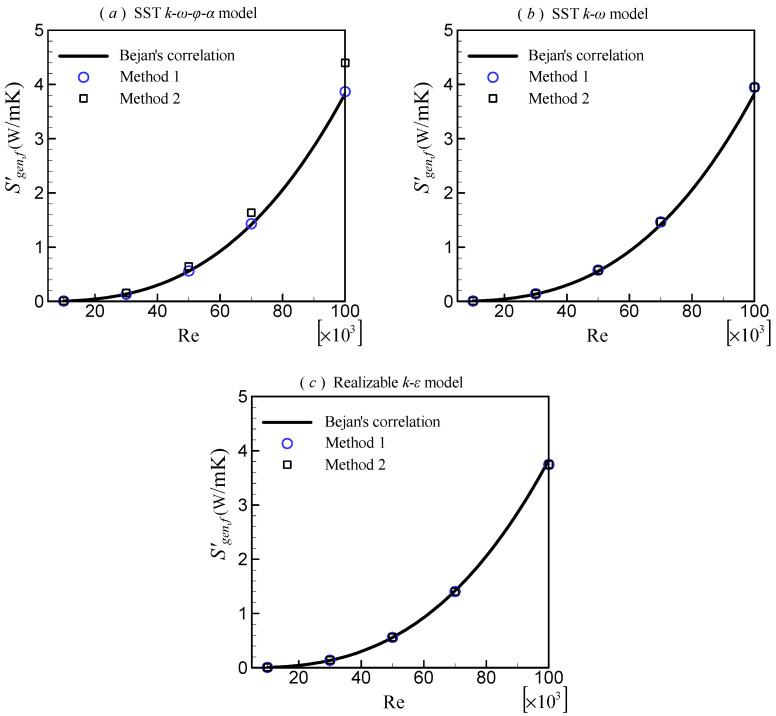
Comparison of different computational methods for$\;S_{gen,f}^{\prime }$.

Figure 7 depicts a comparison of the results obtained by using Methods 1 and 2. Because the observed phenomena are the same for tubes of different diameters, only the results with *A_c_* = 0.000005 m^2^ are provided. The results show that $S_{gen,f}^{\prime }$ increases with the increasing Re. For the SST k–ω–φ–α model, the results predicted by the two methods are significantly different. Compared with the Bejan’s correlation (Equation (19)), the result predicted by Method 1 agrees well, but the result predicted by Method 2 deviates. The difference at Re = 100,000 is about 14%. This is because the equation $\varepsilon =\beta^{*}k\omega$ does not exactly hold in the SST k–ω–φ–α model. Consequently, Method 2 cannot be used to determine $S_{gen,f}^{\prime }$ for this model. However, for the SST *k*–*ω* and realizable *k*–*ε* models, the results predicted by the two methods are almost indistinguishable, and they are both in good agreement with the Bejan’s correlation.

**Figure 8:**
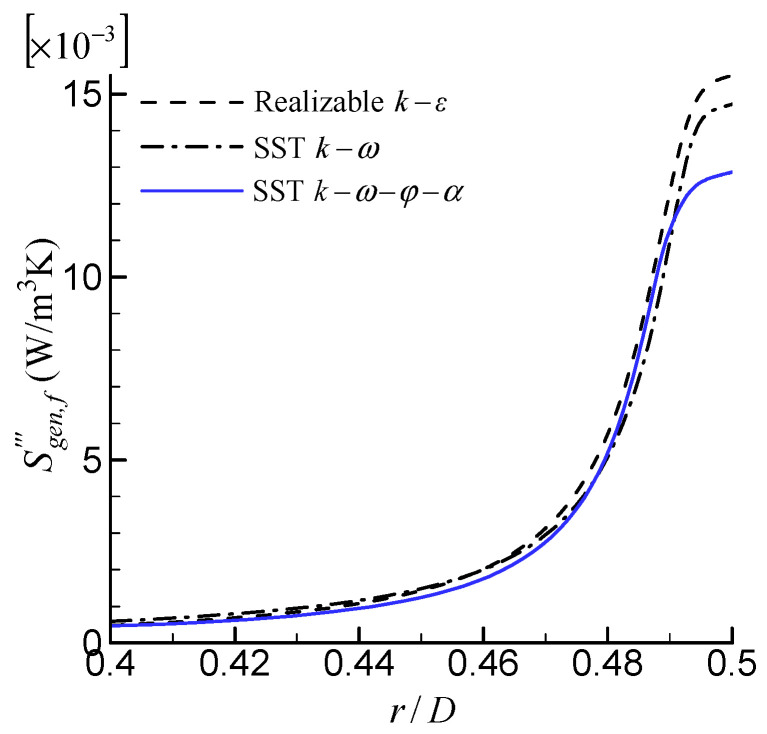
Comparison of local distribution of $\;S_{gen,f}^{\prime \prime \prime }$.

Figure 8 shows the local distribution of the rate of entropy generation per unit volume due to friction irreversibility, $S_{gen,f}^{\prime \prime \prime }$, along the radial direction at Re = 10,000. Similar to $S_{gen,h}^{\prime \prime \prime }$, the $S_{gen,f}^{\prime \prime \prime }$ mainly occurs in the near-wall region. This is because the velocity gradient is large in the near-wall region. The difference among three models occurs in a very thin region near the wall (0.49 < *r*/*D* < 0.5), so that, after being integrated, the difference is small.

As we can see from Equation (19), when the difference in average temperature is small, the $S_{gen,f}^{\prime }$ is inversely proportional to the square of tube diameter at the same Re. This phenomenon is well represented by all three turbulence models. The details are omitted here for brevity.

### 3.4. Rate of Entropy Generation Due to Heat Transfer Irreversibility with Large ΔT

As previously stated, Bejan’s correlation of the rate of entropy generation per unit length due to heat transfer irreversibility ($S_{gen,h}^{\prime }$, Equation (20)) holds only if ΔT*/T_m_* is so small that it can be ignored. In fact, when ΔT*/T_m_* cannot be ignored, $S_{gen,h}^{\prime }$ should be calculated by using the following [3].

(31) $${S^{\prime }}_{gen,h}=\frac{q^{\prime }\Delta T}{T_{ave}(T_{ave}+\Delta T)}.$$

Considering $\Delta T=T_{w}-T_{m}$ and $q^{\prime }=\pi Dq^{\prime \prime }$, using Equations (24) and (25) can yield

(32) $$\Delta T=\frac{q^{\prime }}{\pi \lambda Nu}.$$

**Figure 9:**
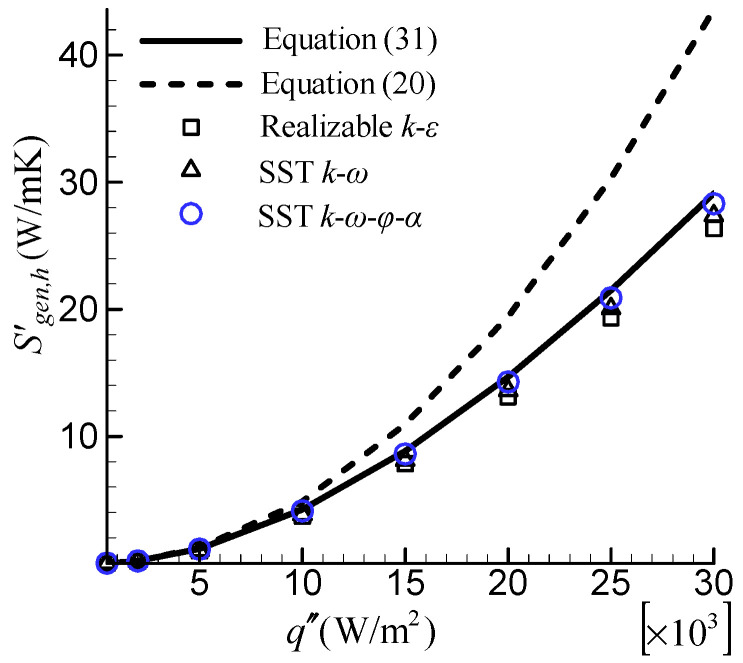
Comparison of the predicted $S_{gen,h}^{\prime }$ by different turbulence models under large wall-bulk temperature difference.

The Bejan’s correlation will deviate significantly in the large tube with a high heat flux and low Re [16]. To investigate the turbulence model’s ability to predict $S_{gen,h}^{\prime }$ when the wall-bulk temperature difference is large, the flow in a large tube with *A_c_* = 0.05 m^2^ and a low Re of 10,000 was considered. Figure 9 compares the predicted $S_{gen,h}^{\prime }$ by different turbulence models with the analytical results. In this case,$\;S_{gen,h}^{\prime }$ increases with increasing heat flux. All three turbulence models’ results agree well with the analytical expression of Equation (31). Among them, the SST k–ω–φ–α model predicts a better result. However, the deviation of the Bejan’s correlation (Equation (20)) is very noticeable and increases with increasing heat flux. The deviation is close to 50% at $q^{\prime \prime }=30,000$ W/m^2^. When examining this case in detail, we discovered that the large wall-bulk temperature difference (exceeding 100 K) is the reason for the large error. This finding suggests that, when using the Bejan’s correlation (Equation (20)) to validate turbulence models, it is critical to ensure that the wall-bulk temperature difference is small enough that ΔT*/T_m_* can be ignored.

### 3.5. Effect of the Temperature-Dependent Fluid Properties

It is well-known that the properties of a fluid are generally temperature-dependent. For example, the viscosity of water is highly temperature-dependent. When the temperature changes significantly, it is inappropriate to consider fluid properties as constants. Ideally, the entropy generation rate will change because of temperature-dependent fluid properties on the flow field. Hence, the effect of temperature-dependent fluid properties on the entropy generation rate was investigated in this subsection. Abbasian Arani and Amani [33] used the following correlations to calculate the water properties.

(33) $$\begin{array}{l} \rho =-764.475639+19.25155\times T-0.07714568\times T^{2}\\ +1.364893\times 10^{-4}\times T^{3}-9.339158\times 10^{-8}\times T^{4} \end{array}.$$

(34) $$\ln\left(\frac{\mu }{0.001792}\right)=-1.24-6.44\times \left(\frac{273.15}{T}\right)+7.68\times {\left(\frac{273.15}{T}\right)}^{2}.$$

(35) $$\begin{array}{l} c_{p}=\text{198,531.690492}-2894.853934\times T+17.2363068\times T^{2}-0.05126994\times T^{3}\\ +7.616133\times 10^{-5}\times T^{4}-4.517821\times 10^{-8}\times T^{5} \end{array}.$$

(36) $$\lambda =-1.549404+0.01553952\times T-3.65967\times 10^{-5}\times T^{2}+2.9401\times 10^{-8}\times T^{3}.$$

**Figure 10:**
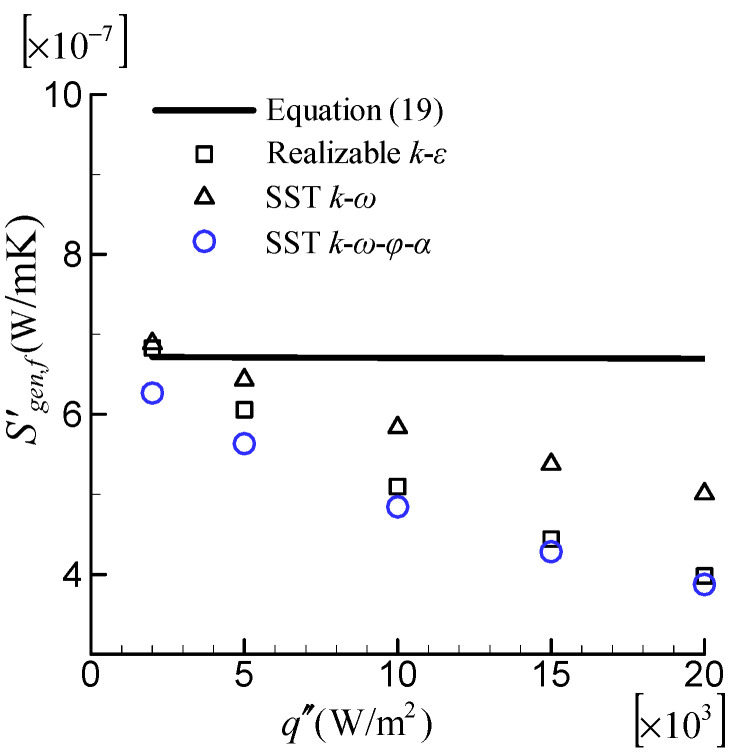
Effect of the temperature-dependent fluid properties on $S_{gen,f}^{\prime }$.

**Figure 11:**
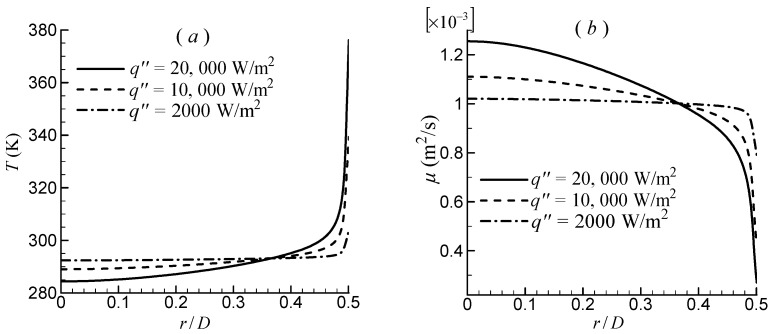
Simulated temperature and fluid molecular viscosity profiles along the lateral section by the SST *k*–*ω*–*φ*–*α* model: (**a**) temperature and (**b**) viscosity.

The flow in the tube with *A_c_* = 0.05 m^2^ at Re = 10,000, which is based on fluid properties at the inlet (*T_in_* = 293.15 K), was computed. Five cases were considered, each with a different heat flux of 2000, 5000, 10,000, 15,000, and 20,000 W/m^2^. Figure 10 depicts the effect of temperature-dependent fluid properties on entropy generation rate due to friction irreversibility, $S_{gen,f}^{\prime }$. The Bejan’s correlation of Equation (19) computed by using constant fluid properties at inlet temperature was included as a reference. The result of Bejan’s correlation decreases only slightly as heat flux increases. Conversely, as the heat flux increases, all $S_{gen,f}^{\prime }$ computed by using CFD results with three turbulence models decreased significantly. The observed behavior can be explained by using detailed flow and temperature field information. Figure 11 depicts the SST k–ω–φ–α model’s simulated temperature and fluid molecular viscosity profiles on the lateral section. The difference in average temperature for different heat fluxes is small. Consequently, the $S_{gen,f}^{\prime }$ predicted by the Bejan’s correlation with constant fluid property, which is only dependent on the average temperature, decreases slightly with increasing heat flux. With increasing heat flux in the near-wall region, the local temperature rises dramatically, significantly lowering the molecular fluid viscosity. Furthermore, there is a high velocity gradient near the wall. Consequently, the $S_{gen,f}^{\prime }$ computed using CFD results (Equation (11)) are significantly reduced. The greater the heat flux, the lower the viscosity and, consequently, the entropy generation.

**Figure 12:**
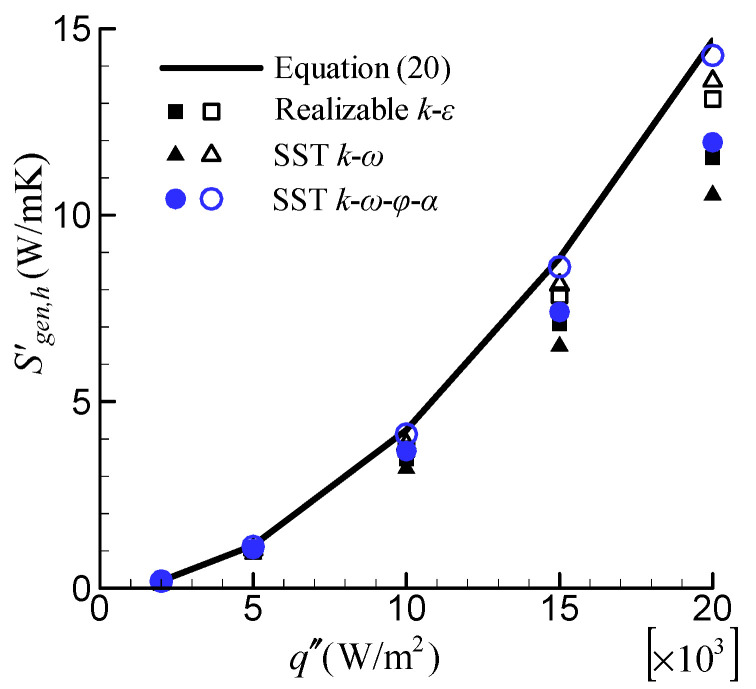
Effect of the temperature-dependent fluid properties on$\;S_{gen,h}^{\prime }$.

The effect of temperature-dependent fluid properties on the rate of entropy generation due to heat-transfer irreversibility, $S_{gen,h}^{\prime }$, is depicted in Figure 12. The CFD predicted $S_{gen,h}^{\prime }$ separately, using temperature-dependent fluid properties (denoted by solid points), and constant fluid properties at inlet temperature (denoted by hollow points) were compared with the Bejan’s correlation of Equation (20). When considering the variation of fluid properties with temperature, $S_{gen,h}^{\prime }$ is significantly reduced. Similar to $S_{gen,f}^{\prime }$, the greater the heat flux applied, the more $S_{gen,h}^{\prime }$ decreases.

This result demonstrates that temperature-dependent fluid properties must be considered when there is a significant temperature change in the flow field. In this case, Bajen’s correlations are inapplicable.

## 4. Conclusions

The rate of entropy generation in a circular tube subjected to uniform heat flux was calculated by using an elliptic blending model (SST k–ω–φ–α). The analytical correlations and results that were computed by using the realizable *k*–*ε* model and the SST *k*–*ω* model were provided as references. As a result of the findings, the following conclusions were reached.

(1) The turbulent Prandtl number influences both the Nusselt number and the entropy generation rate due to heat-transfer irreversibility. For the SST k–ω–φ–α model, Pr_t_ = 0.85 yields the best results. The SST k–ω–φ–α model produces excellent results for all Reynolds numbers; however, both the realizable *k*–*ε* and SST *k*–*ω* models perform poorly for low Reynolds numbers.

(2) The rate of entropy generation due to friction irreversibility was well predicted by all three turbulence models. For the realizable *k*–*ε* and SST *k*–*ω* models, the two methods for calculating $S_{gen,f}^{\prime }\;$led to nearly indistinguishable results. However, For the SST k–ω–φ–α model, the difference between two methods is significant and it is about 14% at Re = 100,000. The method that employs the effective turbulent viscosity, rather than the turbulent dissipation rate, *ε*, should be used.

(3) When the temperature in the flow field varies significantly, the change in fluid properties with a temperature considerably affects entropy generation rates due to friction and heat transfer.

In conclusion, the SST k–ω–φ–α model outperforms the other two turbulence models. Furthermore, although the case studied in this paper is relatively simple, given the inherent advantages of the SST k–ω–φ–α model in complex flows, it has great potential for the application of heat-transfer-system optimization. The application of this model in complex heat-transfer systems (such as separated flow and impinging jet flow) requires continued research and verification.

Finally, it should be pointed out that this paper mainly studied the capabilities of the turbulence model, rather than the detailed behavior of entropy generation rate in circular pipe flow, so we did not focus on the contribution of each part of the entropy generation rate to the total entropy generation rate. In fact, it could be clearly seen that, in small-sized pipe (*A_c_* = 0.000005 m^2^), as shown in Section 3.3 and Section 3.4, the entropy generation rate due to heat transfer is only on the order of 10^−3^ of the entropy generation rate due to friction. Meanwhile, in large-sized pipe (*A_c_* = 0.05 m^2^), as shown in Section 3.5, the entropy generation rate due to friction is only on the order of 10^−7^ of the entropy generation rate due to heat transfer. For the total entropy generation rate, the entropy generation rate due to heat transfer in small-sized pipe and that due to friction in large-sized pipe are completely negligible. Of course, for medium-sized pipes, the entropy generation rates of these two parts are of the same order of magnitude.

## Figures and Tables

**Table 1:** Mesh convergence test (*A_c_* = 0.05 m^2^, Re = 100,000, and *q*″ = 500 W/m^2^).

$\mathrm{Grid}\;(N_{r}\times N_{x})$	*y+*	*f*	*Nu*	$S_{gen,h}^{\prime }\;(W/m\cdot K)$	$S_{gen,f}^{\prime }\;(W/m\cdot K)$
50×100	17.9	6.769 × 10^−2^	4809.66	1.538 × 10^−4^	13.666 × 10^−4^
100×100	1.65	1.991 × 10^−2^	753.14	1.209 × 10^−4^	4.192 × 10^−4^
150×100	0.34	1.818 × 10^−2^	595.42	1.576 × 10^−3^	3.836 × 10^−4^
200×100	0.08	1.809 × 10^−2^	583.33	1.662 × 10^−3^	3.863 × 10^−4^
Ref.	—	1.797 × 10^−2^	598.95	1.621 × 10^−3^	3.840 × 10^−4^

**Table 2:** Material properties for the working fluid (H_2_O) at 293.15 K.

*ρ* (kg/m^3^)	*c_p_* (J/kg·K)	*μ* (Pa·s)	*λ* (W/m·K)
998.2	4182	1.004 × 10^−3^	0.599

**Table 3:** Computational parameters used in each section.

	*A_c_* (m^2^)	*q*″ (W/m^2^)	Re
Section 3.1, Section 3.2 and Section 3.3	0.000005	50,000	10^4^–10^5^
	0.0005	5000	
	0.05	500	
Section 3.4	0.05	500–30,000	10^4^
Section 3.5	0.05	2000–20,000	10^4^

## Data Availability

Not applicable.
